# Supplementary material for: Robust Generation of Ready-to-Use Cryopreserved Motor Neurons from Human Pluripotent Stem Cells for Disease Modeling
Source: Int J Mol Sci. 2022 Nov 3;23(21):13462. doi: 10.3390/ijms232113462 (PMC9657726; doi:10.3390/ijms232113462)
Supplement: Supplementary file 1 [file ijms-23-13462-s001.zip › Supplementary Figure S1.pdf]

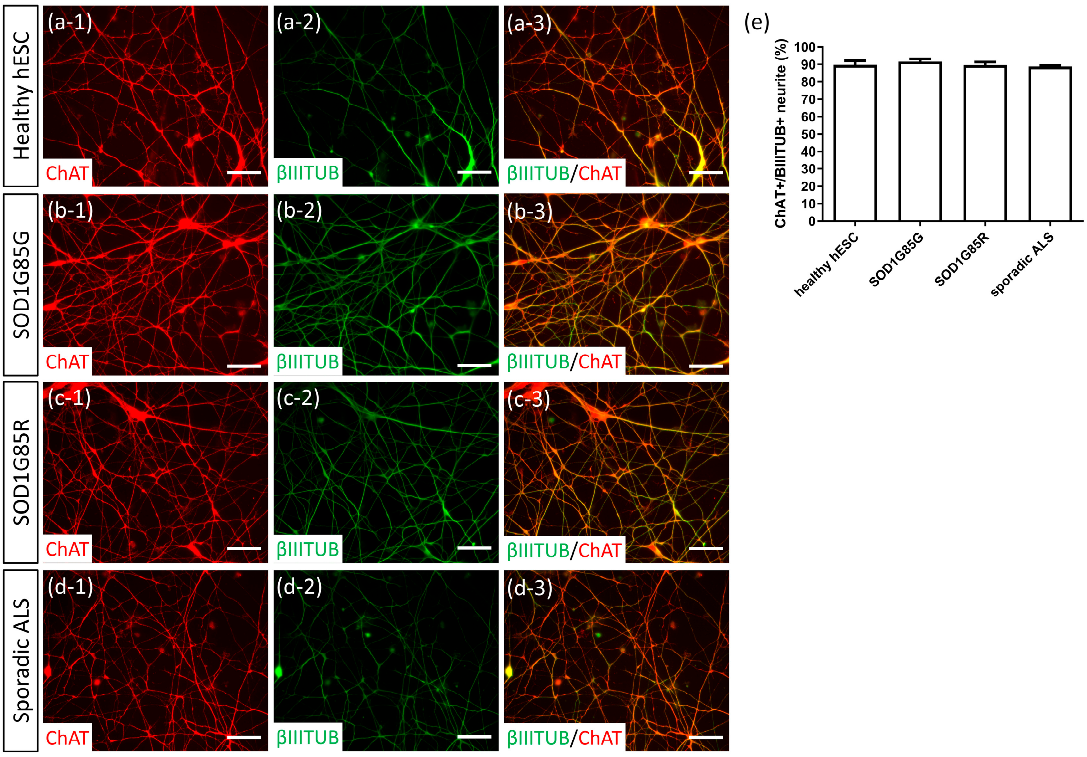

Supplementary Figure S1: The expression of ChAT on thawed MNs. (a–d) Identification H9 healthy hESC (a), $SOD1^{G85G}$ (b), $SOD1^{G85R}$ (c) and sporadic ALS (d) iPSC-derived MNs using MN (ChAT) and neuronal specific marker (β-III-tubulin) expression after 4 days of thawing. (e) Calculation of the ChAT expression ratio of β-III-tubulin+ cells. Number 1–3 presenting the separated and merged images in (a–d). Scale bar, 100 μm.
